# Systems biology graphical notation markup language (SBGNML) version 0.3

**DOI:** 10.1515/jib-2020-0016

**Published:** 2020-06-22

**Authors:** Frank T. Bergmann, Tobias Czauderna, Ugur Dogrusoz, Adrien Rougny, Andreas Dräger, Vasundra Touré, Alexander Mazein, Michael L. Blinov, Augustin Luna

**Affiliations:** BioQUANT/COS, Heidelberg University, INF 267, Heidelberg, 69120, Germany; Faculty of Information Technology, Monash University, Melbourne, Australia; Computer Engineering Department, Bilkent University, Ankara, 06800, Turkey; i-Vis Research Lab, Bilkent University, Ankara, 06800, Turkey; Biotechnology Research Institute for Drug Discovery, AIST, Tokyo, 135-0064, Japan; Computational Bio Big-Data Open Innovation Laboratory (CBBD-OIL), AIST, Tokyo, 169-8555, Japan; Computational Systems Biology of Infection and Antimicrobial-Resistant Pathogens, Institute for Bioinformatics and Medical Informatics (IBMI), Tübingen, 72076, Germany; Department of Computer Science, University of Tübingen, Tübingen, 72076, Germany; German Center for Infection Research (DZIF), Partner Site Tübingen, Tübingen, 72076, Germany; Department of Biology, Norwegian University of Science and Technology (NTNU), Trondheim, Norway; Luxembourg Centre for Systems Biomedicine, University of Luxembourg, Belvaux, L-4367, Luxembourg; Center for Cell Analysis and Modeling, UConn Health, Farmington, CT, 06030, USA; cBio Center, Department of Data Sciences, Dana–Farber Cancer Institute, Boston, MA, 02215, USA; Department of Cell Biology, Harvard Medical School, Boston, MA, 02115, USA; European Institute for Systems Biology and Medicine, CIRI UMR5308, CNRS-ENS-UCBL-INSERM, Université de Lyon, Lyon, 69007, France

**Keywords:** biological process diagrams, network biology; pathway diagram, SBGN, systems biology, visualization

## Abstract

This document defines Version 0.3 Markup Language (ML) support for the Systems Biology Graphical Notation (SBGN), a set of three complementary visual languages developed for biochemists, modelers, and computer scientists. SBGN aims at representing networks of biochemical interactions in a standard, unambiguous way to foster efficient and accurate representation, visualization, storage, exchange, and reuse of information on all kinds of biological knowledge, from gene regulation, to metabolism, to cellular signaling. SBGN is defined neutrally to programming languages and software encoding; however, it is oriented primarily towards allowing models to be encoded using XML, the eXtensible Markup Language. The notable changes from the previous version include the addition of attributes for better specify metadata about maps, as well as support for multiple maps, sub-maps, colors, and annotations. These changes enable a more efficient exchange of data to other commonly used systems biology formats (e. g., BioPAX and SBML) and between tools supporting SBGN (e. g., CellDesigner, Newt, Krayon, SBGN-ED, STON, cd2sbgnml, and MINERVA). More details on SBGN and related software are available at http://sbgn.org. With this effort, we hope to increase the adoption of SBGN in bioinformatics tools, ultimately enabling more researchers to visualize biological knowledge in a precise and unambiguous manner.

***Corresponding author: Augustin Luna**, cBio Center, Department of Data Sciences, Dana–Farber Cancer Institute, Boston, 02215, MA, USA; and Department of Cell Biology, Harvard Medical School, Boston, MA, 02115, USA, E-mail: aluna@jimmy.harvard.edu. https://orcid.org/0000-0001-5709-371X
**Frank T. Bergmann:** BioQUANT/COS, Heidelberg University, INF 267, Heidelberg, 69120, Germany, E-mail: frank.bergmann@bioquant.uni-heidelberg.de. https://orcid.org/0000-0001-5553-4702
**Tobias Czauderna:** Faculty of Information Technology, Monash University, Melbourne, Australia, E-mail: tobias.czauderna@monash.edu. https://orcid.org/0000-0002-1788-9593
**Ugur Dogrusoz:** Computer Engineering Department, Bilkent University, Ankara, 06800, Turkey; i-Vis Research Lab, Bilkent University, Ankara, 06800, Turkey, E-mail: ugur@cs.bilkent.edu.tr. https://orcid.org/0000-0002-7153-0784
**Adrien Rougny:** Biotechnology Research Institute for Drug Discovery, AIST, Tokyo, 135-0064, Japan; Computational Bio Big-Data Open Innovation Laboratory (CBBD-OIL), AIST, Tokyo, 169-8555, Japan, E-mail: rougny.adrien@aist.go.jp. https://orcid.org/0000-0002-2118-035X
**Andreas Dräger:** Computational Systems Biology of Infection and Antimicrobial-Resistant Pathogens, Institute for Bioinformatics and Medical Informatics (IBMI), Tübingen, 72076, Germany; Department of Computer Science, University of Tübingen, Tübingen, 72076, Germany; and German Center for Infection Research (DZIF), Partner Site Tübingen, Tübingen, 72076, Germany, E-mail: andreas.draeger@uni-tuebingen.de. https://orcid.org/0000-0002-1240-5553
**Vasundra Touré:** Department of Biology, Norwegian University of Science and Technology (NTNU), Trondheim, Norway, E-mail: vasundra.toure@ntnu.no. https://orcid.org/0000-0003-4639-4431
**Alexander Mazein:** Luxembourg Centre for Systems Biomedicine, University of Luxembourg, Belvaux, L-4367, Luxembourg; European Institute for Systems Biology and Medicine, CIRI UMR5308, CNRS-ENS-UCBL-INSERM, Université de Lyon, Lyon, 69007, France, E-mail: alexander.mazein@uni.lu. https://orcid.org/0000-0001-7137-4171
**Michael L. Blinov:** Center for Cell Analysis and Modeling, UConn Health, Farmington, CT, 06030, USA, E-mail: blinov@uchc.edu. https://orcid.org/0000-0002-9363-9705

SBGNML Version 0.3 Specification

## SBGN Markup Language (‘sbgnml’)

| | |
|---|---|
| Frank T Bergmann | *BioQUANT/COS, Heidelberg University, DE* |
| Tobias Czauderna | *Monash University, AU* |
| Ugur Dogrusoz | *Bilkent University, TR* |
| Adrien Rougny | *AIST, JP* |
| Andreas Dräger | *Universität Tübingen, DE* |
| Vasundra Touré | *NTNU, NO* |
| Alexander Mazein | *University of Luxembourg, LU* |
| Michael L Blinov | *University of Connecticut, US* |
| Augustin Luna | *Dana-Farber Cancer Institute, US* |

sbgn-editors@googlegroups.com

Version 0.3

April 1, 2020

The latest release, past releases, and other materials related to this specification are available at
`http://sbgn.org`

*This* release of the specification is available at
`http://co.mbine.org/specifications/sbgnml.version-0.3.release-1`

## Contents

**1 Introduction** 3

**2 Package syntax and semantics** 5

2.1 Document conventions . . . 5

2.2 Namespace URI and other declarations necessary for using this package . . . 5

2.3 Primitive data types . . . 6

2.3.1 Type `Language` . . . 6

2.3.2 Type `Class` . . . 6

2.3.3 Type `Orientation` . . . 7

2.3.4 Type `EntityType` . . . 7

2.3.5 Type `ArcGroupType` . . . 7

2.3.6 Type `ArcClass` . . . 8

2.4 The **SBGN** class . . . 8

2.5 The **Map** class . . . 9

2.6 The **Point** class . . . 11

2.7 The **BBox** class . . . 11

2.8 The **Label** class . . . 12

2.9 The **Glyph** class . . . 13

2.10 The **Port** class . . . 16

2.11 The **State** class . . . 17

2.12 The **Callout** class . . . 18

2.13 The **Entity** class . . . 19

2.14 The **Arc** class . . . 20

2.15 The **ArcGroup** class . . . 22

**3 Example SBGN Maps** 24

3.1 Example of a Process Description Map . . . 24

3.2 Example of an Entity Relationship Map . . . 27

3.3 Example of an Activity Flow Map . . . 30

**A Validation of SBGN documents** 32

A.1 Validation and consistency rules . . . 32

A.1.1 Implied Specification Rules . . . 32

A.1.2 Syntax Rules . . . 32

A.1.3 Semantic Rules . . . 32

A.1.4 Layout Rules . . . 32

**B Including color / style information** 33

**C Acknowledgments** 35

**D Financial Support** 36

**References** 37

## 1 Introduction

The Systems Biology Graphical Notation (SBGN) aims to standardize the graphical/visual representation of biochemical and cellular processes (Czauderna and Schreiber, 2017; Junker et al., 2012; Novère et al., 2009; Touré et al., 2018). The goal of SBGN is to represent networks of biochemical interactions in a standard, unambiguous way to foster efficient and accurate representation, visualization, storage, exchange, and reuse of various types of biological knowledge (e.g., gene regulation, metabolism, and cellular signaling). SBGN is defined by comprehensive sets of symbols with precise semantics, together with detailed syntactic rules defining their use and interpretation. Overall, SBGN is made up of three complementary visual languages.

- The SBGN Process Description (PD) language (Rougny et al., 2019) visualizes the temporal courses of the molecular processes and interactions taking place between biochemical entities in a particular system. This type of diagram depicts how entities transition from one form to another as a result of different influences to describe the temporal aspects of a biological system. Nodes describe entity pools (e.g., metabolites, proteins, and complexes) and processes (e.g., associations and influences). The edges describe relationships between the nodes (e.g., consumption and stimulation).
- The SBGN Entity Relationship (ER) language (Sorokin et al., 2015) visualizes the relationships in which a given entity can participate without regard for the temporal aspects. Relationships can be seen as rules describing the influences of entity pool nodes on relationships. Relationships are independent, and this independence is essential in avoiding the combinatorial explosion inherent to process description diagrams. The nodes describe biological entities such as proteins and complexes, and the edges between them describe interactions, relationships and/or influences (e.g., complex formation, stimulation, and inhibition).
- The SBGN Activity Flow (AF) language (Mi et al., 2015) visualizes the influences between the activities displayed by molecular entities, rather than the entities themselves. Nodes in SBGN AF diagrams describe the biological activities of the entities such as protein kinase activity or binding activity. The edges describe influences between the activities (e.g., positive influence and negative influence).

Formal specification describing the visual languages of SBGN, as well as other materials and software, are available from the SBGN project web site, `http://sbgn.org`. The SBGN project seeks a standardized intermediate format—a *lingua franca*—enabling communication of the essential aspects of the visual representations of networks of biochemical interactions.

SBGN is defined neutrally concerning programming languages and software encoding; however, it is oriented primarily towards allowing models to be encoded using XML, the eXtensible Markup Language (Bray et al., 2004). This document contains specifications of how SBGN maps should be serialized in XML. Note that this specification is related to all three SBGN languages, with classes such as Glyph and Arc having the same definition and attributes across all languages. Unlike SBGN, SBGNML does not deal with biological meaning, but, instead, focuses on the computational representation of SBGN graphics, so it is comparable with graphical exchange standards like GraphML[1] and SVG[2].

This document describes Milestone 3 (known as Version 0.3) of SBGNML. The previous version of this work (SBGNML Milestone 2) was released in 2011 (van Iersel et al., 2012). Below is a list of major changes from previous work:

- The ability to store multiple SBGN maps within a single file. An "id" attribute has added as an identifier for individual SBGN maps to disambiguate them.
- The "language" attribute has been deprecated to add a "version" attribute. The value for this attribute is a URI identifier that gives metadata information about the SBGN language, level, and version of the map.
- Complete support for submaps has been implemented with the inclusion of two attributes: "mapRef" and "tagRef".

[1] `http://graphml.graphdrawing.org/`
[2] `https://www.w3.org/Graphics/SVG/`

- The SBGN AF "perturbation" glyph, which was an activity node, has been deprecated and is now a unit of information.
- The support of colors and other annotations through extensions enables the storage of rendering information and biological annotations (e.g., database identifiers).

The definition of the model description language presented here does not specify *how* programs should communicate or read/write SBGN. We assume that for diagram editing software to communicate a model encoded in SBGN, the program will have to translate its internal data structures to and from SBGNML, use a suitable transmission medium and protocol, and to provide any further necessary infrastructure. However, these issues are outside the scope of this document. The software library libSBGN (van Iersel et al., 2012) was developed for reading, writing, and manipulating SBGN maps stored in SBGNML format. A broad set of software tools support SBGNML, including modeling software CellDesigner (Balaur et al., 2020), SBGN editors Newt (Sari et al., 2015), Krayon for SBGN[3], and SBGN-ED (Czauderna et al., 2010). STON (Touré et al., 2016) and ySBGN[4] provide conversion between SBNGML and GraphML/Neo4j, respectively. The software EscherConverter provides an SBGN viewer and a bidirectional converter for metabolic maps in JSON format and SBGNML (King et al., 2015). Numerous databases (Reactome (Croft et al., 2011), Panther Pathways (Mi et al., 2017), Pathways Commons (Rodchenkov et al., 2020), PathWhiz (Pon et al., 2015), Path2Models (Büchel et al., 2013), MetaCrop (Schreiber et al., 2012) and Atlas of Cancer Signaling Networks (Kuperstein et al., 2015)) provide SBGNML export.

[3] https://github.com/draeger-lab/krayon4sbgn
[4] https://github.com/sbgn/ySBGN

## 2 Package syntax and semantics

### 2.1 Document conventions

We use Unified Modeling Language (UML) version 2.0 (Dennis et al. 2015) class diagram notation to define the constructs provided by this package. We first provide an overall view of the various data types and constructs along with their relationships, followed by a more local view of the constructs and their relationships in associated sections.

In this section, we define the syntax and semantics of the Systems Biology Graphical Notation - Markup Language. We expound on the various data types and constructs defined in this package, then in Section 3 on page 24; we provide complete examples of using the constructs in sample SBGN models.

### 2.2 Namespace URI and other declarations necessary for using this package

SBGNML is identified uniquely by an XML namespace URI. An SBGN document must declare the following is the namespace URI for this version of the Systems Biology Graphical Notation - Markup Language for SBGNML version 0.3:

“`http://sbgn.org/libsbgn/0.3`”

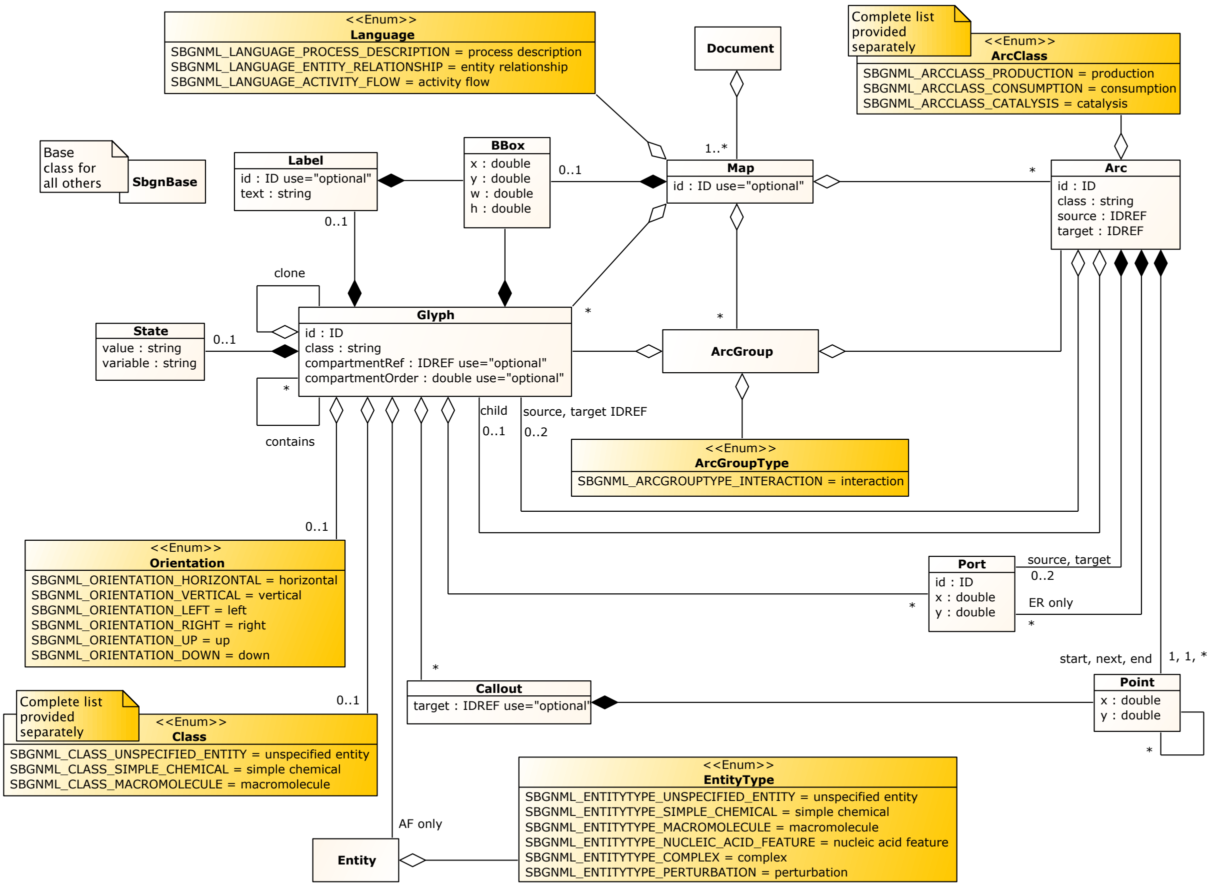

***Figure 1:*** *A UML representation of the Systems Biology Graphical Notation - Markup Language. See Section 2.1 for conventions related to this figure.*

### 2.3 Primitive data types

Section 3.1 of the SBML Level 3 specification (Hucka et al., 2019) defines several primitive data types and also uses XML Schema 1.0 data types (Biron and Malhotra, 2000). We assume and use some of them in the rest of this specification, particularly `float`, `ID`, `IDREF`, and `string`. The Systems Biology Graphical Notation - Markup Language defines other primitive types as described below.

#### *2.3.1 Type* `Language`

<<Enum>>
**Language**

SBGNML_LANGUAGE_PROCESS_DESCRIPTION = process description
SBGNML_LANGUAGE_ENTITY_RELATIONSHIP = entity relationship
SBGNML_LANGUAGE_ACTIVITY_FLOW = activity flow

***Figure 2:*** *A UML representation of the* ***Language*** *type for the Systems Biology Graphical Notation - Markup Language. See Section 2.1 for conventions related to this figure.*

The `Language` is an enumeration of values used to specify which SBGN Language is encoded on the **Map** element.

The possible values are `process description`, `entity relationship`, and `activity flow`.

#### *2.3.2 Type* `Class`

<<Enum>>
**Class**

SBGNML_CLASS_UNSPECIFIED_ENTITY = unspecified entity
SBGNML_CLASS_SIMPLE_CHEMICAL = simple chemical
SBGNML_CLASS_MACROMOLECULE = macromolecule
SBGNML_CLASS_NUCLEIC_ACID_FEATURE = nucleic acid
SBGNML_CLASS_SIMPLE_CHEMICAL_MULTIMER = simple chemical
SBGNML_CLASS_MACROMOLECULE_MULTIMER = macromolecule
SBGNML_CLASS_NUCLEIC_ACID_FEATURE_MULTIMER = nucleic acid feature
SBGNML_CLASS_COMPLEX = complex;SBGNML_CLASS_COMPLEX_MULTIMER = complex multimer
SBGNML_CLASS_SOURCE_AND_SINK = source and sink
SBGNML_CLASS_PERTURBATION = perturbation
SBGNML_CLASS_BIOLOGICAL_ACTIVITY = biological activity
SBGNML_CLASS_PERTURBING_AGENT = perturbing agent
SBGNML_CLASS_COMPARTMENT = compartment
SBGNML_CLASS_SUBMAP = submap
SBGNML_CLASS_TAG = tag;SBGNML_CLASS_TERMINAL = terminal
SBGNML_CLASS_PROCESS = process
SBGNML_CLASS_OMITTED_PROCESS = omitted process
SBGNML_CLASS_UNCERTAIN_PROCESS = uncertain process
SBGNML_CLASS_ASSOCIATION = association
SBGNML_CLASS_DISSOCIATION = dissociation
SBGNML_CLASS_PHENOTYPE = phenotype
SBGNML_CLASS_AND = and
SBGNML_CLASS_OR = or
SBGNML_CLASS_NOT = not
SBGNML_CLASS_STATE_VARIABLE = state variable
SBGNML_CLASS_UNIT_OF_INFORMATION = unit of information
SBGNML_CLASS_ENTITY = entity
SBGNML_CLASS_OUTCOME = outcome
SBGNML_CLASS_INTERACTION = interaction
SBGNML_CLASS_INFLUENCE_TARGET = influence target
SBGNML_CLASS_ANNOTATION = annotation
SBGNML_CLASS_VARIABLE_VALUE = variable value
SBGNML_CLASS_IMPLICIT_XOR = implicit xor
SBGNML_CLASS_DELAY = delay
SBGNML_CLASS_EXISTENCE = existence
SBGNML_CLASS_LOCATION = location
SBGNML_CLASS_CARDINALITY = cardinality
SBGNML_CLASS_OBSERVABLE = observable

***Figure 3:*** *A UML representation of the* ***Class*** *type for the Systems Biology Graphical Notation - Markup Language. See Section 2.1 for conventions related to this figure.*

The `Class` is an enumeration of values used to specify what type a **Glyph** is encoding.

The possible values are `unspecified entity`, `simple chemical`, `macromolecule`, `nucleic acid feature`, `simple chemical multimer`, `macromolecule multimer`, `nucleic acid feature multimer`, `complex`, `complex multimer`, `source and sink`, `perturbation`, `biological activity`, `perturbing agent`, `compartment`, `submap`, `tag`, `terminal`, `process`, `omitted process`, `uncertain process`, `association`, `dissociation`, `phenotype`, `and`, `or`, `not`, `equivalence`, `state variable`, `unit of information`, `entity`, `outcome`, `interaction`, `influence target`, `annotation`, `variable value`, `implicit xor`, `delay`, `existence`, `location`, `cardinality`, and `observable`.

#### 2.3.3 Type `Orientation`

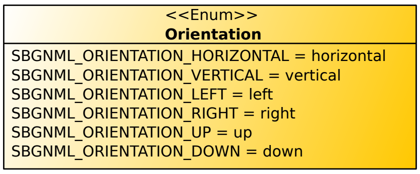

***Figure 4:** A UML representation of the **Orientation** type for the Systems Biology Graphical Notation - Markup Language. See Section 2.1 for conventions related to this figure.*

The `Orientation` is an enumeration of values used to express how to draw asymmetric glyphs.

The orientation of Process Nodes is either "horizontal" or "vertical". It refers to an (imaginary) line connecting the two in/out sides of the PN.

The possible values are `horizontal`, `vertical`, `left`, `right`, `up`, and `down`. The value refers to the direction at which the arrow side of the glyph is pointing.

#### 2.3.4 Type `EntityType`

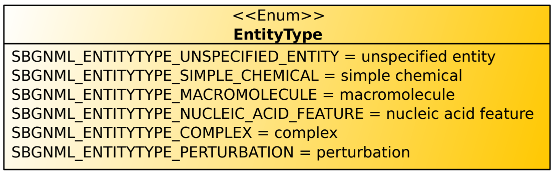

***Figure 5:** A UML representation of the **EntityType** type for the Systems Biology Graphical Notation - Markup Language. See Section 2.1 for conventions related to this figure.*

The `EntityType` is an enumeration of values used for Activity Flow maps that specifies the auxiliary unit to display.

The possible values are `unspecified entity`, `simple chemical`, `macromolecule`, `nucleic acid feature`, and `complex`.

#### 2.3.5 Type `ArcGroupType`

The `ArcGroupType` is an enumeration of values used to define the semantic of an **ArcGroup**.

The only possible value is `interaction`.

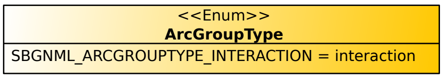

***Figure 6:*** *A UML representation of the* ***ArcGroupType*** *type for the Systems Biology Graphical Notation - Markup Language. See Section 2.1 for conventions related to this figure.*

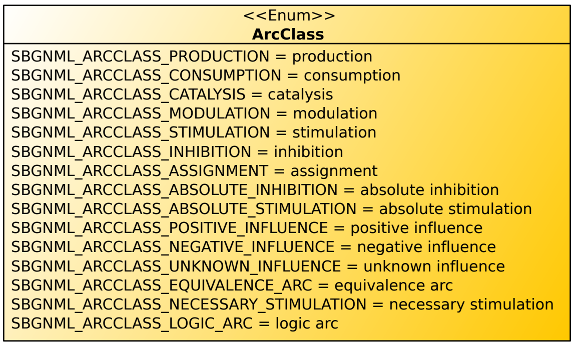

***Figure 7:*** *A UML representation of the* ***ArcClass*** *type for the Systems Biology Graphical Notation - Markup Language. See Section 2.1 for conventions related to this figure.*

#### *2.3.6 Type* `ArcClass`

The `ArcClass` is an enumeration of values used to define the semantic of an **Arc**.

The possible values are `production`, `consumption`, `catalysis`, `modulation`, `stimulation`, `inhibition`, `assignment`, `absolute inhibition`, `absolute stimulation`, `positive influence`, `negative influence`, `unknown influence`, `equivalence arc`, `necessary stimulation`, and `logic arc`.

### 2.4 The SBGN class

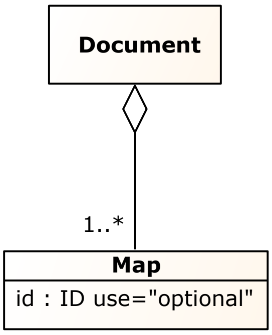

***Figure 8:*** *A UML representation of the* ***Document*** *class for the Systems Biology Graphical Notation - Markup Language. See Section 2.1 for conventions related to this figure.*

The **Document** object shown in Figure 8 corresponds to the XML element `sbgn`. The `sbgn` element is the root of any SBGNML document.

The **Document** object derives from the ***SbgnBase*** class and thus inherits all attributes and elements that are present for this class. A **Document** contains one or more **Map** elements.

***Example***

The following example shows an `sbgn` element definition.

```
<sbgn ...>
    ...
</sbgn>
```

### 2.5 The Map class

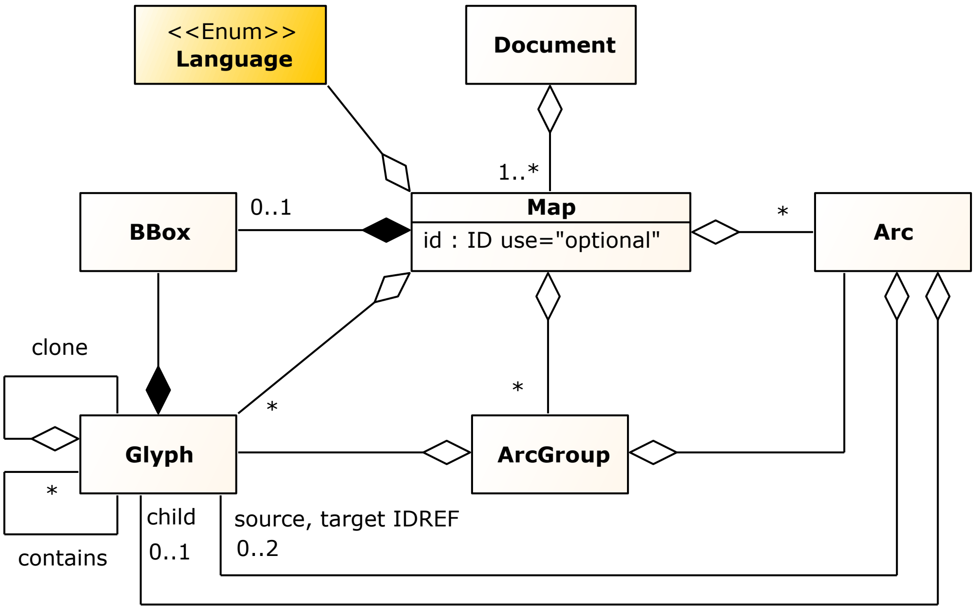

***Figure 9:*** *A UML representation of the **Map** class for the Systems Biology Graphical Notation - Markup Language. See* *Section 2.1* *for conventions related to this figure.*

The `map` element describes a single SBGN map.

The **Map** object derives from the ***SbgnBase*** class and thus inherits all attributes and elements that are present for this class. A **Map** contains exactly one **BBox** element.

A **Map** may contain one or more:

- **Glyph** elements.
- **Arc** elements.
- **ArcGroup** elements.

In addition, the **Map** object has the following attributes.

***The* `id` *attribute***

A **Map** has an optional attribute `id` of type `ID`.

#### *The* `language` *attribute*

A **Map** has an optional attribute `language` of type string. While the type is of `string`, the values should be one of the ones defined in `Language`, i.e., one of the following:

- `process description`
- `entity relationship`
- `activity flow`

The `language` attribute has been deprecated as of Version 0.3, in favor of the `version` attribute. One of the attributes has to be defined on a map element.

#### *The* `version` *attribute*

A **Map** has an optional attribute `version` of type `URI` with the URL to the SBGN language and version it is referring to. The attribute can take one of the following values:

- http://identifiers.org/combine.specifications/sbgn.pd.level-1.version-2.0
- http://identifiers.org/combine.specifications/sbgn.pd.level-1.version-1.3
- http://identifiers.org/combine.specifications/sbgn.pd.level-1.version-1.2
- http://identifiers.org/combine.specifications/sbgn.pd.level-1.version-1.1
- http://identifiers.org/combine.specifications/sbgn.pd.level-1.version-1.0
- http://identifiers.org/combine.specifications/sbgn.pd.level-1.version-1
- http://identifiers.org/combine.specifications/sbgn.er.level-1.version-2
- http://identifiers.org/combine.specifications/sbgn.er.level-1.version-1.2
- http://identifiers.org/combine.specifications/sbgn.er.level-1.version-1.1
- http://identifiers.org/combine.specifications/sbgn.er.level-1.version-1.0
- http://identifiers.org/combine.specifications/sbgn.er.level-1.version-1
- http://identifiers.org/combine.specifications/sbgn.af.level-1.version-1.2
- http://identifiers.org/combine.specifications/sbgn.af.level-1.version-1.0
- http://identifiers.org/combine.specifications/sbgn.af.level-1.version-1

The `version` attribute should be used in favor of the `language` attribute. One of the attributes has to be defined on a map element.

#### *Example*

The following example shows an abbreviated SBGN **Map** definition within an sbgn element definition. The example shows a **Map** with a `version` attribute.

```
<sbgn ...>
    ...
    <map id="m1" version="http://identifiers.org/combine.specifications/sbgn.pd.level-1.version-1.3">
        ...
    </map>
    ...
</sbgn>
```

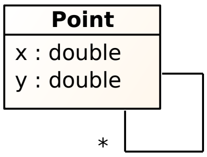

***Figure 10:*** *A UML representation of the* ***Point*** *class for the Systems Biology Graphical Notation - Markup Language. See Section 2.1 for conventions related to this figure.*

### 2.6 The Point class

The **Point** object encodes `x` and `y` coordinates.

The origin is located in the top-left corner of the map.

There is no unit: proportions must be preserved, but the maps can be drawn at any scale. In the example test files, to obtain a drawing similar to the reference file, values in the corresponding file should be read as pixels.

Additionally, it may contain zero, one, or two child **Point** objects, which can be used to encode quadratic or cubic Bézier points.

The **Point** object derives from the ***SbgnBase*** class and thus inherits all attributes and elements that are present for this class. In addition, the **Point** object has the following attributes.

##### *The `x` attribute*

A **Point** has a required attribute `x` of type `double`. It represents the Cartesian `x` coordinate horizontally, increasing from left to right.

##### *The `y` attribute*

A **Point** has a required attribute `y` of type `double`. It represents the Cartesian `y` coordinate vertically, increasing from top to bottom.

##### *Example*

The following example shows a **Point** definition within an abbreviated SBGN map definition. The example shows a **Point** on a **Callout**.

```
<map ...>
    ...
    <glyph ...>
        ...
        <callout ...>
            <point x="100.0" y="200.0"/>
        </callout>
        ...
    </glyph>
    ...
</map>
```

### 2.7 The BBox class

**BBox** encodes the bounding box of its parent element.

The **BBox** object derives from the ***SbgnBase*** class and thus inherits all attributes and elements that are present for this class. In addition, the **BBox** object has the following attributes.

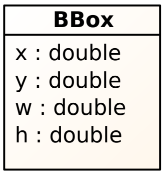

***Figure 11:*** *A UML representation of the* ***BBox*** *class for the Systems Biology Graphical Notation - Markup Language. See* *Section 2.1* *for conventions related to this figure.*

##### *The* `x` *attribute*

A **BBox** has a required attribute `x` of type `double`. It represents the Cartesian `x` coordinate horizontally, increasing from left to right.

##### *The* `y` *attribute*

A **BBox** has a required attribute `y` of type `double`. It represents the Cartesian `y` coordinate vertically, increasing from top to bottom.

##### *The* `w` *attribute*

A **BBox** has a required attribute `w` of type `double`. It encodes the width of the bounding box.

##### *The* `h` *attribute*

A **BBox** has a required attribute `h` of type `double`. It encodes the height of the bounding box.

##### *Example*

The following example shows a **BBox** definition within an abbreviated SBGN map definition. The example shows the **BBox** on a **Glyph**.

```
<map ...>
    ...
    <glyph ...>
        ...
        <bbox x="10.0" y="10.0" w="100.0" h="50.0"/>
        ...
    </glyph>
    ...
</map>
```

### 2.8 The Label class

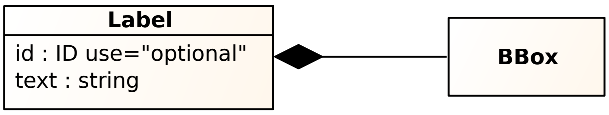

***Figure 12:*** *A UML representation of the* ***Label*** *class for the Systems Biology Graphical Notation - Markup Language. See* *Section 2.1* *for conventions related to this figure.*

The **Label** element describes the text accompanying a glyph.

The **Label** object derives from the ***SbgnBase*** class and thus inherits all attributes and elements that are present for this class. A **Label** may contain exactly one **BBox** element. In addition, the **Label** object has the following attributes.

#### *The `id` attribute*

A **Label** has an optional attribute `id` of type `ID`.

#### *The `text` attribute*

A **Label** has a required attribute `text` of type `string`. The text element is a simple string. Multi-line labels are allowed. Line breaks are encoded as `
` as specified by the XML standard.

#### *The BBox element of a Label*

The `bbox` element of a label is optional. When no bounding box is defined, the bounding box of the parent glyph is inherited. The label should be drawn centered horizontally and vertically in the bounding box.

When the bounding box is inherited, the label may spill outside (just like it can spill outside its parent glyph).

An explicit `bbox` provides more definite information regarding what surface the label should cover. It defines an upper boundary outside of which the label should (ideally) not spill. It also represents a preferred size: the surface covered by the label can be smaller, but should ideally be as close as possible to the bounding box.

In most glyph classes (EPNs, unit of information, etc.), the label is supposed to be centered, so the bounding box is usually omitted (unless there is a specific hint to be shared concerning the area the label should ideally cover).

However, the label of a compartment or a complex can be drawn anywhere inside the glyph, so these should preferably have an explicit bounding box.

#### *Example*

The following example shows a **Label** definition within an abbreviated SBGN map definition. The example shows the **Label** without a `bbox` element on a **Glyph**.

```
<map ...>
    ...
    <glyph ...>
        ...
        <label text="label"/>
        ...
    </glyph>
    ...
</map>
```

### 2.9 The Glyph class

The glyph element is:

- either a stand-alone, high-level SBGN node (an EPN, PN, compartment, etc.)
- or a sub-node (state variable, unit of information, inside of a complex, etc.)

In the first case, it is a child of the map element.

In the second case, it is a child of another glyph element.

The **Glyph** object derives from the ***SbgnBase*** class and thus inherits all attributes and elements that are present for this class.

A **Glyph** contains:

- exactly one **BBox** element that describes the bounding box of the glyph.

A **Glyph** may contain:

- exactly one **Label** element that describes the text accompanying the glyph.

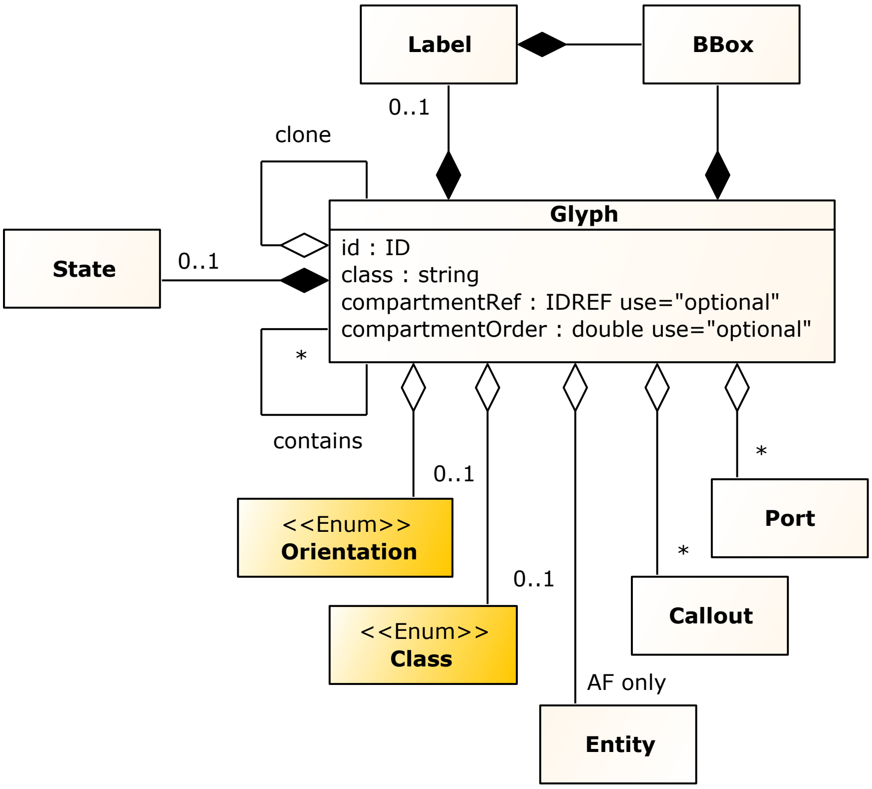

***Figure 13:*** *A UML representation of the* ***Glyph*** *class for the Systems Biology Graphical Notation - Markup Language. See Section 2.1 for conventions related to this figure.*

- exactly one **State** element that carries the information of a state variable.
- exactly one **Glyph** element called "clone", indicating that the Glyph carries a clone marker. The label element of the child glyph can be used to place text in the clone marker. Figure 15 shows an example.
- exactly one **Callout** element. The callout element is only used for glyphs of class `annotation`. It contains the coordinate of the point where the annotation points to, as well as a reference to the element that is pointed to.
- exactly one **Entity** element. The entity is only used in Activity Flow maps. It can only be used on a unit of information glyph on a biological activity glyph, where it is compulsory. It is used to indicate the shape of this unit of information.
- zero or more child **Glyph** elements. These will be, for example, used by glyphs of class `complex` and hold the individual components.
- zero or more child **Port** elements describing the anchor points for this glyph.

In addition, the **Glyph** object has the following attributes.

##### *The* `id` *attribute*

A **Glyph** has a required attribute `id` of type `ID`. The `id` attribute (`xsd:ID`) of a glyph can be referred to, e.g., as a source by arc elements, a target by arc elements or callout elements, by other glyphs if the glyph is of the class `compartment`.

The `xsd:ID` type is an alphanumeric identifier, starting with a letter.

It is recommended to generate meaningless IDs (e.g., “`glyph1234`”) and avoid IDs with meaning (e.g., “`epn_ethanol`”)

#### *The* `class` *attribute*

A **Glyph** has a required attribute `class` of type `string`. While the type is of `string`, the values should be one of the ones defined in `Class`.

#### *The* `compartmentRef` *attribute*

A **Glyph** has an optional attribute `compartmentRef` of type `IDREF`.

The `compartmentRef` is a reference to the ID of the compartment that this glyph is part of. It should only be used if there is at least one explicit compartment present on the map. Compartments are only used in PD and AF, and thus this attribute as well. For PD, this should be used only for EPNs.

In case there are no compartments, entities that can have a location, such as EPNs, are implicit members of an invisible compartment that encompasses the whole map. In that case, this attribute must be omitted.

#### *The* `compartmentOrder` *attribute*

A **Glyph** has an optional attribute `compartmentOrder` of type `double`.

The `compartmentOrder` attribute can be used to define a drawing order for compartments. It enables tools to draw compartments in the correct order, especially in the case of overlapping compartments. Compartments are only used in PD and AF, and, thus, this attribute as well.

The attribute is of type `float`, and the attribute value has not to be unique.

Compartments with higher `compartmentOrder` are drawn on top. The attribute is optional and should only be used for compartments.

#### *The* `orientation` *attribute*

A **Glyph** has an optional attribute `orientation` of type string. While the type is of `string`, the values should be one of the ones defined in `Orientation`. The `orientation` attribute is used to express how to draw asymmetric glyphs.

The orientation of Process Nodes is either `horizontal` or `vertical`. It refers to an (imaginary) line connecting the two in/out sides of the PN.

The orientation of Tags can be `left`, `right`, `up`, or `down`. It refers to the direction at which the arrow side of the glyph is pointing.

#### *Example*

The following example shows a **Glyph** definition within an abbreviated SBGN map definition. The example shows a **Glyph** of class `macromolecule` with an optional attribute `compartmentRef`. Figure 14 shows the corresponding visual representation.

```
<map ...>
    ...
    <glyph id="glyph1" class="macromolecule" compartmentRef="glyphcomp1">
        <label text="Glucose"/>
        <bbox x="10.0" y="10.0" w="100.0" h="50.0"/>
    </glyph>
    ...
</map>
```

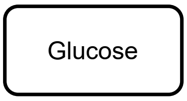

***Figure 14:*** *Visual representation of a* ***Glyph*** *of the class* `macromolecule`.

#### *Example Clone Marker*

The following example shows a **Glyph** definition within an abbreviated SBGN map definition. The example shows a **Glyph** of class `macromolecule` with an optional attribute `compartmentRef` and a clone marker. Figure 15 shows the corresponding visual representation.

```
<map ...>
    ...
    <glyph id="glyph1" class="macromolecule" compartmentRef="glyphcomp1">
        <clone>
            <label text="marker">
                <bbox x="10.0" y="47.5" w="100.0" h="12.5"/>
            </label>
        </clone>
        <label text="Glucose"/>
        <bbox x="10.0" y="10.0" w="100.0" h="50.0"/>
    </glyph>
    ...
</map>
```

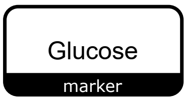

***Figure 15:*** *Visual representation of a* ***Glyph*** *of the class* `macromolecule` *with a clone marker.*

### 2.10 The Port class

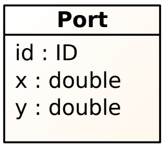

***Figure 16:*** *A UML representation of the* ***Port*** *class for the Systems Biology Graphical Notation - Markup Language. See Section 2.1 for conventions related to this figure.*

A port element describes an anchor point, which arc elements can refer to as a source or target. It consists of absolute 2D Cartesian coordinates and a unique id attribute.

Two port elements are required for process nodes and logical operators ( `and`, `or`, `not`, and `equivalence`). They represent the extremity of the two “arms” which protrude on both sides of the core of the glyph (= square or circle shape).

The **Port** object derives from the ***SbgnBase*** class and, thus, inherits all attributes and elements that are present for this class. In addition, the **Port** object has the following attributes.

##### *The `id` attribute*

A **Port** has a required attribute `id` of type `ID`.

##### *The `x` attribute*

A **Point** has a required attribute `x` of type `double`. It represents the Cartesian `x` coordinate horizontally, increasing from left to right.

##### *The `y` attribute*

A **Point** has a required attribute `y` of type `double`. It represents the Cartesian `y` coordinate vertically, increasing from top to bottom.

##### *Example*

The following example shows a **Port** definition within an abbreviated SBGN map definition. The example shows two **Port**s on a **Glyph**.

```
<map ...>
    ...
    <glyph id="glyph1" ...>
        ...
        <port id="glyph1.1" x="100.0" y="100.0"/>
        <port id="glyph1.2" x="120.0" y="100.0"/>
        ...
    </glyph>
    ...
</map>
```

### 2.11 The State class

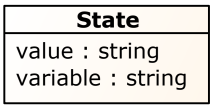

***Figure 17:*** *A UML representation of the* ***State*** *class for the Systems Biology Graphical Notation - Markup Language. See Section 2.1 for conventions related to this figure.*

The state element should only be used for state variables. It replaces the label element used for other glyphs. It describes the text to be drawn inside the state variable.

The **State** object derives from the ***SbgnBase*** class and thus inherits all attributes and elements that are present for this class. In addition, the **State** object has the following attributes.

##### *The `variable` attribute*

A **State** has an optional attribute `variable` of type `string`. It describes the site where the modification described by the value attribute occurs. It is:

- optional when there is only one state variable on the parent EPN
- required when there is more than one state variable on the parent EPN

##### *The `value` attribute*

A **State** has an optional attribute `value` of type `string`. It represents the state of the variable. It can be:

- either from a predefined set of strings (e.g., “P”, “S”, etc.) which correspond to specific SBO terms (cf. SBGN specifications)
- or any arbitrary `string`.

***Example***

The following example shows a **State** definition within an abbreviated SBGN map definition. The example depicts two **State**s on a **Glyph** of the class `macromolecule`, one **State** with a `value` attribute and a `variable` attribute and one **State** with a `variable` attribute only. Figure 18 shows the corresponding visual representation.

```
<map ...>
    ...
    <glyph id="glyph1" class="entity">
        <label text="CaMKII"/>
        <bbox x="10.0" y="20.0" w="140.0" h="50.0"/>
        <glyph id="glyph1a" class="state variable">
            <state variable="T286" value="P"/>
            <bbox x="13.5" y="11.0" w="63.0" h="18.0"/>
        </glyph>
        <glyph id="glyph1b" class="state variable">
            <state variable="T306"/>
            <bbox x="93.5" y="11.0" w="43.0" h="18.0"/>
        </glyph>
    </glyph>
    ...
</map>
```

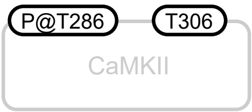

***Figure 18:*** *Visual representation of two **States** on a **Glyph** of the class* `macromolecule`.

### 2.12 The Callout class

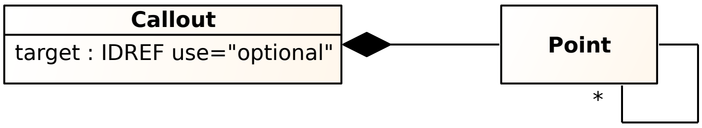

***Figure 19:*** *A UML representation of the **Callout** class for the Systems Biology Graphical Notation - Markup Language. See Section 2.1 for conventions related to this figure.*

Callouts are used in the case of glyphs of class `annotation`. The callout is always optional. It can be used to show which element the callout points to.

The **Callout** object derives from the ***SbgnBase*** class and thus inherits all attributes and elements that are present for this class. A **Callout** contains at most one **Point** element. In addition, the **Callout** object has the following attributes.

##### *The `target` attribute*

A **Callout** has an optional attribute `target` of type `IDREF`. If specified, it references either a **Glyph** or an **Arc** in the **Map**.

##### *Example*

The following example shows a **Callout** definition within an abbreviated SBGN map definition. The example depicts a **Callout** on a **Glyph** of class `annotation`, pointing to a **Glyph** of the class `macromolecule`. Figure 20 contains the corresponding visual representation.

```
<map ...>
    ...
    <glyph id="g1" class="macromolecule">
        <label text="label"/>
        <bbox x="10.0" y="95.0" w="100.0" h="50.0"/>
    </glyph>
    <glyph id="g2" class="annotation">
        <callout target="g1">
            <point x="95.0" y="110.0"/>
        </callout>
        <label text="annotation"/>
        <bbox x="95.0" y="10.0" w="100.0" h="100.0"/>
    </glyph>
    ...
</map>
```

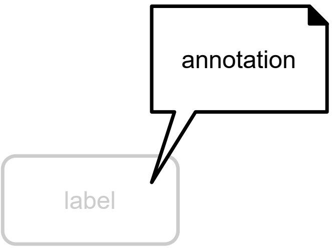

***Figure 20:*** *Visual representation of a **Callout** on a **Glyph** of class `annotation` pointing to a **Glyph** of the class `macromolecule`.*

### 2.13 The Entity class

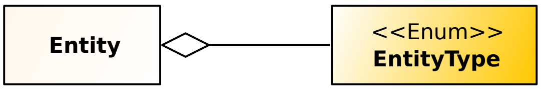

***Figure 21:*** *A UML representation of the **Entity** class for the Systems Biology Graphical Notation - Markup Language. See Section 2.1 for conventions related to this figure.*

An entity is only used in Activity Flow maps. It should be placed on a unit of information subglyph of an activity glyph and is used to indicate the entity that performs the activity.

The **Entity** object derives from the ***SbgnBase*** class and thus inherits all attributes and elements that are present for this class. In addition, the **Entity** object has the following attributes.

#### *The* `name` *attribute*

An **Entity** has a required attribute `name` of type `string`.

#### *Example*

The following example shows an **Entity** definition within an abbreviated SBGN map definition. The example shows an **Entity** with the `name` “macromolecule” placed on a **Glyph** of class `biological activity`. Figure 22 shows the corresponding visual representation.

```
<map ...>
    ...
    <glyph id="glyph1" class="biological activity">
        <label text="Activity"/>
        <bbox x="10.0" y="20.0" w="100.0" h="50.0"/>
        <glyph id="glyph1a" class="unit of information">
            <label text="Entity"/>
            <entity name="macromolecule"/>
            <bbox x="13.0" y="11.0" w="44.0" h="18.0"/>
        </glyph>
    </glyph>
    ...
</map>
```

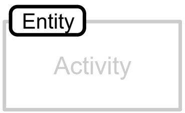

***Figure 22:*** *Visual representation of an* ***Entity*** *on a* ***Glyph*** *of the class* `biological activity`.

### 2.14 The Arc class

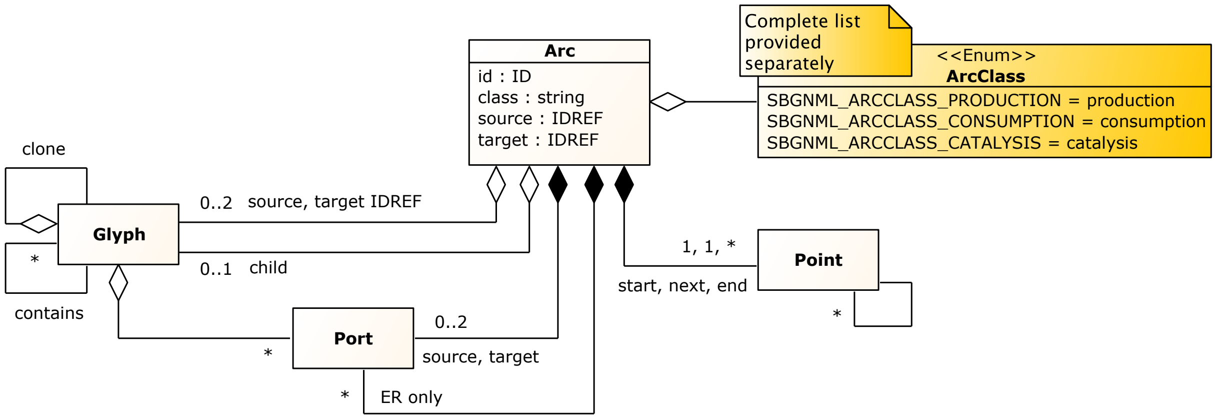

***Figure 23:*** *A UML representation of the* ***Arc*** *class for the Systems Biology Graphical Notation - Markup Language. See Section 2.1 for conventions related to this figure.*

The arc element describes an SBGN arc between two SBGN nodes. It contains:

- For PD maps: an optional stoichiometry marker,

- For ER maps: an optional cardinality marker (e.g., “cis” or “trans”), zero or more ports (influence targets), and zero or more outcomes,
- a mandatory source and target (glyph or port),
- a geometric description of its whole path from start to end. This path can involve any number of straight lines or quadratic/cubic Bézier curves.

The **Arc** object derives from the ***SbgnBase*** class and thus inherits all attributes and elements that are present for this class.

An **Arc** can contain zero or more child **Glyph** elements. These can be a stoichiometry marker (PD maps), a cardinality marker (ER maps), or outcome glyphs (ER maps).

An **Arc** contains at the very least one **Point** element with an element named `start` that represents the start point of the arc, and another **Point** element with element name `end` as the endpoint. Additionally, it may contain any number of **Point** elements with element name `next` that represent bend points along the way from start to end.

An **Arc** may also contain any number of **Port** elements.

In addition, the **Arc** object has the following attributes.

#### *The `id` attribute*

An **Arc** has a required attribute `id` of type `ID`.

#### *The `class` attribute*

An **Arc** has a required attribute `class` of type `string`. It describes what kind of an **Arc** this element represents. While the data type is of `string`, the values ought to be from the `ArcClass` enumeration.

#### *The `source` attribute*

An **Arc** has a required attribute `source` of type `IDREF`. It specifies the source element for this arc.

#### *The `target` attribute*

An **Arc** has a required attribute `target` of type `IDREF`. It specifies the target element for this arc.

#### *Example*

The following example shows an **Arc** definition within an abbreviated SBGN map definition. The example shows one **Arc** of class `consumption` and one **Arc** of class `production`. Figure 24 shows the corresponding visual representation.

```
<map ...>
    ...
    <arc id="arc1" class="consumption" source="glyph1" target="glyph2.1">
        <start x="50.0" y="25.0"/>
        <end x="100.0" y="25.0"/>
    </arc>
    ...
    <arc id="arc2" class="production" source="glyph2.2" target="glyph3">
        <start x="140.0" y="25.0"/>
        <end x="190.0" y="25.0"/>
    </arc>
    ...
</map>
```

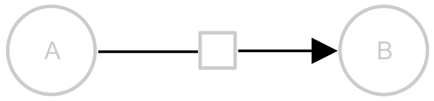

***Figure 24:*** *Visual representation of an* ***Arc*** *of class* `consumption` *(left) and an* ***Arc*** *of class* `production` *(right).*

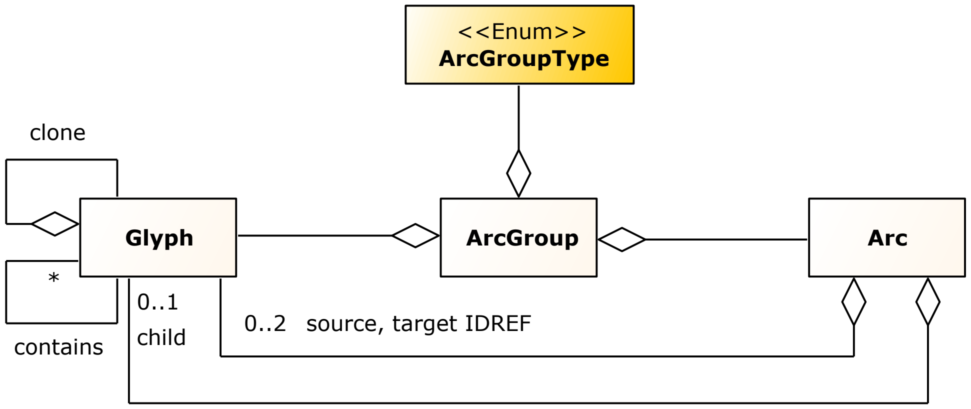

***Figure 25:*** *A UML representation of the* ***ArcGroup*** *class for the Systems Biology Graphical Notation - Markup Language. See Section 2.1 for conventions related to this figure.*

### 2.15 The ArcGroup class

The arc group describes a set of arcs and glyphs that have a relation together, for example, in ER arcs of class `interaction` around a glyph of class `interaction`.

Note that, despite the name, an arc group contains both arcs and glyphs.

The **ArcGroup** object derives from the ***SbgnBase*** class and thus inherits all attributes and elements that are present for this class.

An **ArcGroup** can contain:

- zero or more child **Glyph** elements,
- zero or more child **Arc** elements.

In addition, the **ArcGroup** object has the following attributes.

##### *The* `class` *attribute*

An **ArcGroup** has a required attribute `class` of type `string`. While the type is of `string`, the values should be one of the ones defined in `ArcGroupType`.

##### *The Glyph element of an ArcGroup*

An **ArcGroup** can contain **Glyph**s. For example, an **ArcGroup** of class `interaction` must contain one **Glyph** of class `interaction` representing the circle of an $n$-ary interaction. The glyph itself can contain one or more child **Glyph**s of the class `outcome`. Please note, the specification for the Entity Relationship language does not define a **Glyph** node of class `interaction`. It is only introduced here to represent the circle mentioned above.

##### *The Arc element of an ArcGroup*

An **ArcGroup** can have multiple **Arc**s. They are all assumed to form a single hyperarc-like structure.

#### *Example*

The following example shows an **ArcGroup** definition within an abbreviated SBGN map definition. The example shows an **ArcGroup** of class `interaction` with the **Glyph** of class `interaction` and two **Arc**s of class `interaction`. The glyph contains one child, a **Glyph** of the class `outcome`. Figure 26 shows the corresponding visual representation.

```
<map ...>
    ...
    <arcgroup class="interaction">
        <glyph id="glyph1" class="interaction">
            <bbox x="180.0" y="17.5" w="35.0" h="35.0"/>
            <glyph id="glyph1a" class="outcome">
                <bbox x="191.5" y="46.5" w="12.0" h="12.0"/>
            </glyph>
        </glyph>
        <arc id="arc1" class="interaction" source="glyph1" target="glyph2">
            <start x="180.0" y="35.0"/>
            <end x="110.0" y="35.0"/>
        </arc>
        <arc id="arc2" class="interaction" source="glyph1" target="glyph3">
            <start x="215.0" y="35.0"/>
            <end x="285.0" y="35.0"/>
        </arc>
    </arcgroup>
    ...
</map>
```

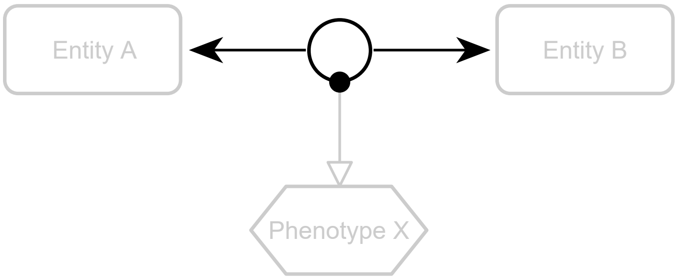

***Figure 26:*** *Visual representation of an* ***ArcGroup*** *of class* `interaction` *with one* ***Glyph*** *of class* `interaction` *and two* ***Arc****s.*

## 3 Example SBGN Maps

This section provides complete examples, one for each SBGN language, showing how to use the elements described in the previous section, in sample SBGN maps.

### 3.1 Example of a Process Description Map

The following example of a Process Description map shows a gene-regulatory network – the activated STAT1$\alpha$ induction of the IRF1 gene. Figure 27 shows the corresponding visual representation of the Process Description map.

```
<?xml version="1.0" encoding="UTF-8" standalone="yes"?>
<sbgn xmlns="http://sbgn.org/libsbgn/0.3">
    <map id="map1" language="process description">
        <glyph id="glyph0" class="complex">
            <bbox x="5.0" y="2.5" w="180.0" h="105.0"/>
            <glyph id="glyph1" class="macromolecule">
                <label text="STAT1$\alpha$"/>
                <bbox x="25.0" y="25.0" w="140.0" h="60.0"/>
                <glyph id="glyph1a" class="unit of information">
                    <label text="mt:prot"/>
                    <bbox x="36.0" y="16.5" w="48.0" h="17.0"/>
                </glyph>
            </glyph>
        </glyph>
        <glyph id="glyph2" class="nucleic acid feature">
            <label text="IRF1-GAS"/>
            <bbox x="235.0" y="25.0" w="120.0" h="60.0"/>
            <glyph id="glyph2a" class="unit of information">
                <label text="ct:grr"/>
                <bbox x="244.5" y="16.5" w="41.0" h="17.0"/>
            </glyph>
        </glyph>
        <glyph id="glyph3" class="nucleic acid feature">
            <label text="IRF1"/>
            <bbox x="465.0" y="430.0" w="120.0" h="60.0"/>
            <glyph id="glyph3a" class="unit of information">
                <label text="ct:mRNA"/>
                <bbox x="496.5" y="421.5" w="57.0" h="17.0"/>
            </glyph>
        </glyph>
        <glyph id="glyph4" class="and" orientation="vertical">
            <bbox x="375.0" y="365.0" w="40.0" h="40.0"/>
            <port id="glyph4.2" x="395.0" y="425.0"/>
            <port id="glyph4.1" x="395.0" y="345.0"/>
        </glyph>
        <glyph id="glyph5" class="process">
            <bbox x="515.0" y="540.0" w="20.0" h="20.0"/>
            <port id="glyph5.1" x="505.0" y="550.0"/>
            <port id="glyph5.2" x="545.0" y="550.0"/>
        </glyph>
        <glyph id="glyph6" class="process">
            <bbox x="385.0" y="450.0" w="20.0" h="20.0"/>
            <port id="glyph6.1" x="375.0" y="460.0"/>
            <port id="glyph6.2" x="415.0" y="460.0"/>
        </glyph>
        <glyph id="glyph7" class="source and sink">
            <bbox x="425.0" y="530.0" w="40.0" h="40.0"/>
        </glyph>
        <glyph id="glyph8" class="source and sink">
            <bbox x="295.0" y="440.0" w="40.0" h="40.0"/>
        </glyph>
```

```
<glyph id="glyph9" class="association" orientation="vertical">
    <bbox x="165.0" y="150.0" w="20.0" h="20.0"/>
    <port id="glyph9.2" x="175.0" y="180.0"/>
    <port id="glyph9.1" x="175.0" y="140.0"/>
</glyph>
<glyph id="glyph13" class="complex">
    <bbox x="65.0" y="207.5" w="220.0" h="215.0"/>
    <glyph id="glyph11" class="complex">
        <bbox x="85.0" y="222.5" w="180.0" h="105.0"/>
        <glyph id="glyph10" class="macromolecule">
            <label text="STAT1$\alpha$"/>
            <bbox x="105.0" y="245.0" w="140.0" h="60.0"/>
            <glyph id="glyph10a" class="unit of information">
                <label text="mt:prot"/>
                <bbox x="151.0" y="236.5" w="48.0" h="17.0"/>
            </glyph>
        </glyph>
    </glyph>
    <glyph id="glyph12" class="nucleic acid feature">
        <label text="IRF1-GAS"/>
        <bbox x="115.0" y="350.0" w="120.0" h="60.0"/>
        <glyph id="glyph12a" class="unit of information">
            <label text="ct:grr"/>
            <bbox x="154.5" y="341.5" w="41.0" h="17.0"/>
        </glyph>
    </glyph>
</glyph>
<glyph id="glyph14" class="nucleic acid feature">
    <label text="IRF1"/>
    <bbox x="455.0" y="275.0" w="120.0" h="60.0"/>
    <glyph id="glyph14a" class="unit of information">
        <label text="ct:gene"/>
        <bbox x="489.5" y="266.5" w="51.0" h="17.0"/>
    </glyph>
</glyph>
<glyph id="glyph15" class="macromolecule">
    <label text="IRF1"/>
    <bbox x="590.0" y="520.0" w="120.0" h="60.0"/>
    <glyph id="glyph15a" class="unit of information">
        <label text="mt:prot"/>
        <bbox x="626.0" y="511.5" w="48.0" h="17.0"/>
    </glyph>
</glyph>
<arc id="arc0" class="necessary stimulation" source="glyph4.2" target="glyph6">
    <start x="395.0" y="425.0"/>
    <end x="395.0" y="450.0"/>
</arc>
<arc id="arc1" class="necessary stimulation" source="glyph3" target="glyph5">
    <start x="525.0" y="490.0"/>
    <end x="525.0" y="540.0"/>
</arc>
<arc id="arc2" class="consumption" source="glyph8" target="glyph6.1">
    <start x="335.0" y="460.0"/>
    <end x="375.0" y="460.0"/>
</arc>
<arc id="arc3" class="production" source="glyph6.2" target="glyph3">
    <start x="415.0" y="460.0"/>
    <end x="465.0" y="460.0"/>
</arc>
<arc id="arc4" class="consumption" source="glyph7" target="glyph5.1">
    <start x="465.0" y="550.0"/>
    <end x="505.0" y="550.0"/>
</arc>
<arc id="arc5" class="production" source="glyph5.2" target="glyph15">
    <start x="545.0" y="550.0"/>
    <end x="590.0" y="550.0"/>
```

```
        </arc>
        <arc id="arc6" class="production" source="glyph9.2" target="glyph13">
            <start x="175.0" y="180.0"/>
            <end x="175.0" y="207.5"/>
        </arc>
        <arc id="arc7" class="logic arc" source="glyph14" target="glyph4.1">
            <start x="455.0" y="325.0"/>
            <end x="395.0" y="345.0"/>
        </arc>
        <arc id="arc8" class="logic arc" source="glyph13" target="glyph4.1">
            <start x="285.0" y="330.0"/>
            <end x="395.0" y="345.0"/>
        </arc>
        <arc id="arc9" class="consumption" source="glyph0" target="glyph9.1">
            <start x="144.5" y="107.5"/>
            <end x="175.0" y="140.0"/>
        </arc>
        <arc id="arc10" class="consumption" source="glyph2" target="glyph9.1">
            <start x="252.5" y="85.0"/>
            <end x="175.0" y="140.0"/>
        </arc>
    </map>
</sbgn>
```

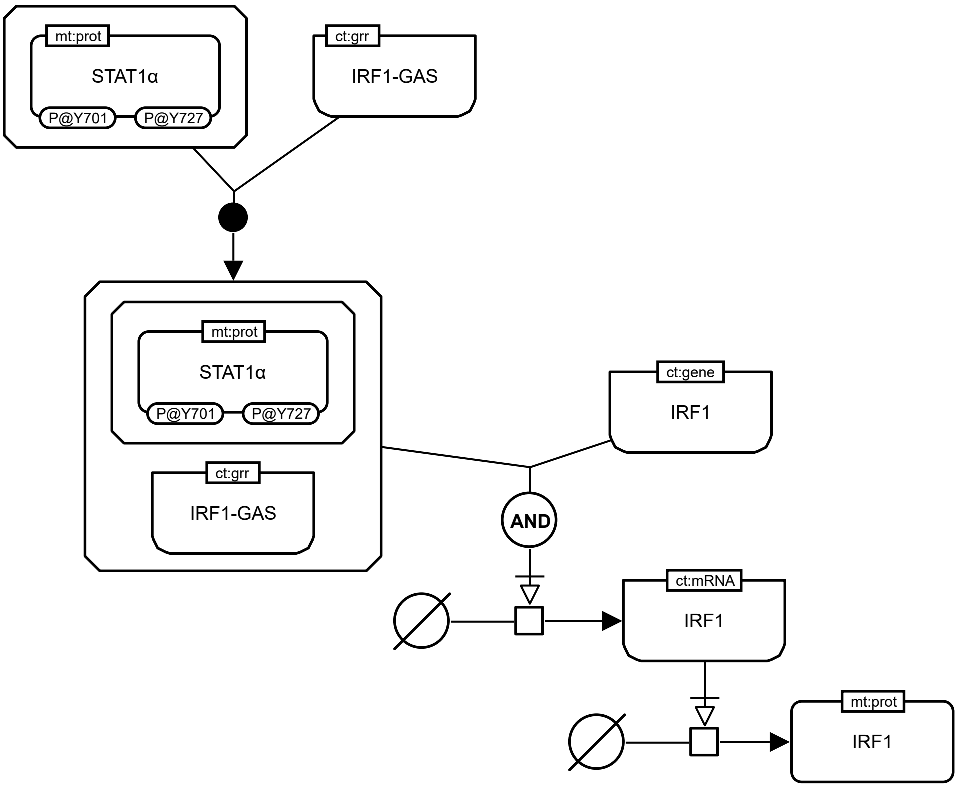

***Figure 27:*** *Activated STAT1α induction of the IRF1 gene.*

### 3.2 Example of an Entity Relationship Map

The following example of an Entity Relationship map shows the principle of the Polymerase Chain Reaction (PCR) (Mullis et al., 1986). Figure 28 shows the corresponding visual representation of the Entity Relationship map.

```
<?xml version="1.0" encoding="UTF-8" standalone="yes"?>
<sbgn xmlns="http://sbgn.org/libsbgn/0.3">
    <map id="map1" language="entity relationship">
        <glyph id="glyph0" class="entity">
            <label text="5' primer"/>
            <bbox x="361.0" y="435.0" w="108.0" h="60.0"/>
            <glyph id="glyph0a" class="unit of information">
                <label text="mt:dna"/>
                <bbox x="391.0" y="487.0" w="48.0" h="16.0"/>
            </glyph>
        </glyph>
        <glyph id="glyph1" class="entity">
            <label text="Antisense"/>
            <bbox x="361.0" y="135.0" w="108.0" h="60.0"/>
            <glyph id="glyph1a" class="unit of information">
                <label text="mt:dna"/>
                <bbox x="391.0" y="127.0" w="48.0" h="16.0"/>
            </glyph>
            <glyph id="glyph1b" class="existence">
                <bbox x="377.0" y="182.5" w="22.0" h="25.0"/>
            </glyph>
        </glyph>
        <glyph id="glyph2" class="entity">
            <label text="3' primer"/>
            <bbox x="61.0" y="435.0" w="108.0" h="60.0"/>
            <glyph id="glyph2a" class="unit of information">
                <label text="mt:dna"/>
                <bbox x="91.0" y="487.0" w="48.0" h="16.0"/>
            </glyph>
        </glyph>
        <glyph id="glyph3" class="entity">
            <label text="Sense"/>
            <bbox x="61.0" y="135.0" w="108.0" h="60.0"/>
            <glyph id="glyph3a" class="unit of information">
                <label text="mt:dna"/>
                <bbox x="91.0" y="127.0" w="48.0" h="16.0"/>
            </glyph>
            <glyph id="glyph3b" class="existence">
                <bbox x="131.0" y="182.5" w="22.0" h="25.0"/>
            </glyph>
        </glyph>
        <glyph id="glyph4" class="perturbing agent">
            <label text="Heat"/>
            <bbox x="211.0" y="5.0" w="108.0" h="60.0"/>
        </glyph>
        <glyph id="glyph5" class="or" orientation="vertical">
            <bbox x="244.0" y="271.0" w="42.0" h="42.0"/>
            <port id="glyph5.1" x="265.0" y="250.0"/>
            <port id="glyph5.2" x="265.0" y="334.0"/>
        </glyph>
        <glyph id="glyph6" class="variable value">
            <label text="T"/>
            <bbox x="378.0" y="275.0" w="20.0" h="20.0"/>
        </glyph>
        <glyph id="glyph7" class="variable value">
            <label text="T"/>
            <bbox x="132.0" y="275.0" w="20.0" h="20.0"/>
        </glyph>
        <arc id="arc0" class="interaction" source="glyph3" target="glyph1">
            <glyph id="arc0.0" class="outcome">
                <bbox x="219.0" y="159.0" w="12.0" h="12.0"/>
```

```
            </glyph>
            <port id="arc0.1" x="265.0" y="165.0"/>
            <glyph id="arc0.2" class="outcome">
                <bbox x="299.0" y="159.0" w="12.0" h="12.0"/>
            </glyph>
            <start x="169.0" y="165.0"/>
            <end x="361.0" y="165.0"/>
        </arc>
        <arc id="arc1" class="interaction" source="glyph2" target="glyph3">
            <glyph id="arc1.0" class="outcome">
                <bbox x="109.0" y="379.0" w="12.0" h="12.0"/>
            </glyph>
            <glyph id="arc1.1" class="outcome">
                <bbox x="109.0" y="329.0" w="12.0" h="12.0"/>
            </glyph>
            <port id="arc1.2" x="115.0" y="285.0"/>
            <port id="arc1.3" x="115.0" y="245.0"/>
            <start x="115.0" y="435.0"/>
            <end x="115.0" y="195.0"/>
        </arc>
        <arc id="arc2" class="interaction" source="glyph0" target="glyph1">
            <glyph id="arc2.0" class="outcome">
                <bbox x="409.0" y="359.0" w="12.0" h="12.0"/>
            </glyph>
            <glyph id="arc2.1" class="outcome">
                <bbox x="409.0" y="329.0" w="12.0" h="12.0"/>
            </glyph>
            <port id="arc2.2" x="415.0" y="285.0"/>
            <port id="arc2.3" x="415.0" y="245.0"/>
            <start x="415.0" y="435.0"/>
            <end x="415.0" y="195.0"/>
        </arc>
        <arc id="arc3" class="assignment" source="glyph7" target="glyph3">
            <port id="arc3.0" x="142.0" y="245.0"/>
            <start x="139.75" y="275.0"/>
            <end x="142.0" y="200.0"/>
        </arc>
        <arc id="arc4" class="assignment" source="glyph6" target="glyph1">
            <port id="arc4.0" x="388.0" y="245.0"/>
            <start x="390.25" y="275.0"/>
            <end x="388.0" y="200.0"/>
        </arc>
        <arc id="arc5" class="absolute inhibition" source="glyph4" target="arc0.1">
            <start x="265.0" y="65.0"/>
            <end x="265.0" y="165.0"/>
        </arc>
        <arc id="arc6" class="absolute inhibition" source="glyph5.1" target="arc0.1">
            <start x="265.0" y="250.0"/>
            <end x="265.0" y="165.0"/>
        </arc>
        <arc id="arc7" class="absolute inhibition" source="glyph4" target="arc2.2">
            <start x="273.4" y="65.0"/>
            <next x="279.0" y="85.0"/>
            <next x="525.0" y="85.0"/>
            <next x="525.0" y="285.0"/>
            <end x="415.0" y="285.0"/>
        </arc>
        <arc id="arc8" class="absolute inhibition" source="arc0.2" target="arc2.3">
            <start x="305.0" y="159.0"/>
            <next x="305.0" y="95.0"/>
            <next x="515.0" y="95.0"/>
            <next x="515.0" y="245.0"/>
            <end x="415.0" y="245.0"/>
        </arc>
        <arc id="arc9" class="necessary stimulation" source="arc2.0" target="arc3.0">
            <start x="409.0" y="365.0"/>
```

```
            <next x="185.0" y="365.0"/>
            <next x="185.0" y="245.0"/>
            <end x="142.0" y="245.0"/>
        </arc>
        <arc id="arc10" class="necessary stimulation" source="arc1.0" target="arc4.0">
            <start x="121.0" y="385.0"/>
            <next x="345.0" y="385.0"/>
            <next x="345.0" y="245.0"/>
            <end x="388.0" y="245.0"/>
        </arc>
        <arc id="arc11" class="absolute inhibition" source="glyph4" target="arc1.2">
            <start x="256.6" y="65.0"/>
            <next x="251.0" y="85.0"/>
            <next x="5.0" y="85.0"/>
            <next x="5.0" y="285.0"/>
            <end x="115.0" y="285.0"/>
        </arc>
        <arc id="arc12" class="absolute inhibition" source="arc0.0" target="arc1.3">
            <start x="225.0" y="159.0"/>
            <next x="225.0" y="95.0"/>
            <next x="15.0" y="95.0"/>
            <next x="15.0" y="245.0"/>
            <end x="115.0" y="245.0"/>
        </arc>
        <arc id="arc13" class="logic arc" source="arc1.1" target="glyph5.2">
            <start x="121.0" y="335.0"/>
            <end x="265.0" y="334.0"/>
        </arc>
        <arc id="arc14" class="logic arc" source="arc2.1" target="glyph5.2">
            <start x="409.0" y="335.0"/>
            <end x="265.0" y="334.0"/>
        </arc>
    </map>
</sbgn>
```

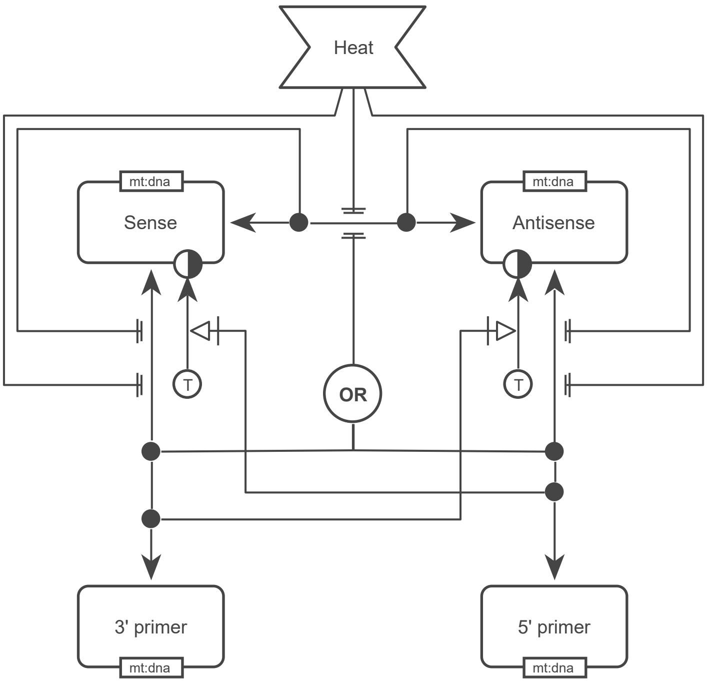

***Figure 28:*** *Principle of the Polymerase Chain Reaction (PCR) (Mullis et al., 1986).*

### 3.3 Example of an Activity Flow Map

The following example of an Activity Flow map shows a signaling pathway involving the regulation of TGFβ-induced metastasis, as described by Adorno et al. (2009). Figure 29 shows the corresponding visual representation of the Activity Flow map.

```
<?xml version="1.0" encoding="UTF-8" standalone="yes"?>
<sbgn xmlns="http://sbgn.org/libsbgn/0.3">
    <map id="map1" language="activity flow">
        <glyph id="glyph0" class="phenotype">
            <label text="Pro-invasion&#xA;migration&#xA;metastasis&#xA;gene expression&#xA;platform"/>
            <bbox x="235.0" y="187.5" w="130.0" h="75.0"/>
        </glyph>
        <glyph id="glyph1" class="biological activity">
            <label text="Metastatic&#xA;suppressor&#xA;genes&#xA;activity"/>
            <bbox x="246.0" y="380.0" w="108.0" h="60.0"/>
        </glyph>
        <glyph id="glyph2" class="biological activity">
            <label text="TGF beta"/>
            <bbox x="246.0" y="5.0" w="108.0" h="60.0"/>
        </glyph>
        <glyph id="glyph3" class="biological activity">
            <label text="Mutant p53/&#xA;P-Smad"/>
            <bbox x="6.0" y="205.0" w="108.0" h="60.0"/>
        </glyph>
        <glyph id="glyph4" class="biological activity">
            <label text="p63"/>
            <bbox x="6.0" y="380.0" w="108.0" h="60.0"/>
        </glyph>
        <glyph id="glyph5" class="biological activity">
            <label text="Ras"/>
            <bbox x="4.0" y="5.0" w="72.0" h="40.0"/>
        </glyph>
        <glyph id="glyph6" class="and" orientation="vertical">
            <bbox x="39.0" y="124.0" w="42.0" h="42.0"/>
            <port id="glyph6.1" x="60.0" y="103.0"/>
            <port id="glyph6.2" x="60.0" y="187.0"/>
        </glyph>
        <arc id="arc0" class="positive influence" source="glyph2" target="glyph0">
            <start x="300.0" y="65.0"/>
            <end x="300.0" y="187.5"/>
        </arc>
        <arc id="arc1" class="positive influence" source="glyph6.2" target="glyph3">
            <start x="60.0" y="187.0"/>
            <end x="60.0" y="205.0"/>
        </arc>
        <arc id="arc2" class="necessary stimulation" source="glyph4" target="glyph1">
            <start x="114.0" y="410.0"/>
            <end x="246.0" y="410.0"/>
        </arc>
        <arc id="arc3" class="logic arc" source="glyph5" target="glyph6.1">
            <start x="45.0" y="45.0"/>
            <end x="60.0" y="103.0"/>
        </arc>
        <arc id="arc4" class="negative influence" source="glyph3" target="glyph4">
            <start x="60.0" y="265.0"/>
            <end x="60.0" y="380.0"/>
        </arc>
        <arc id="arc5" class="negative influence" source="glyph1" target="glyph0">
            <start x="300.0" y="380.0"/>
            <end x="300.0" y="262.5"/>
        </arc>
        <arc id="arc6" class="logic arc" source="glyph2" target="glyph6.1">
            <start x="246.0" y="35.0"/>
            <next x="160.0" y="35.0"/>
```

```
            <end x="60.0" y="103.0"/>
        </arc>
    </map>
</sbgn>
```

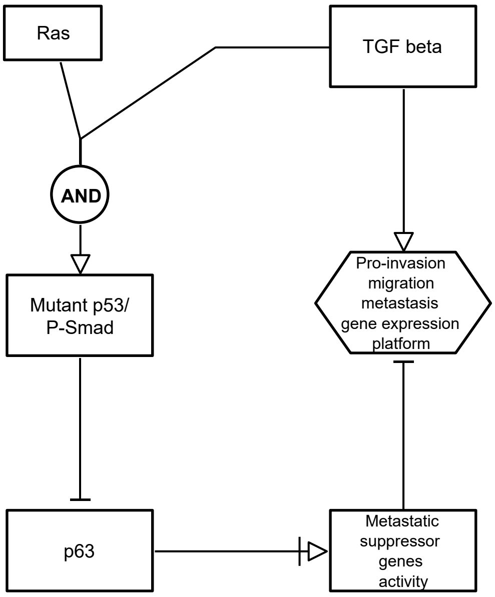

***Figure 29:*** *Regulation of TGFβ–induced metastasis as described by Adorno et al. (2009).*

## A Validation of SBGN documents

### A.1 Validation and consistency rules

This section summarizes the conditions that must (or in some cases, at least *should*) be true of an SBGN document that uses the Systems Biology Graphical Notation - Markup Language. There are different degrees of rule strictness. Formally, the differences are expressed in the statement of a rule: either a rule states that a condition *must* be true, or a rule states that it *should* be true. Rules of the former kind are strict SBGN validation rules—a model encoded in SBGN must conform to all of them in order to be considered valid. Rules of the latter kind are consistency rules.

#### *A.1.1 Implied Specification Rules*

The rest of this specification document implies basic validation rules. Unless explicitly stated, all validation rules concern objects and their attributes defined specifically in the Systems Biology Graphical Notation - Markup Language.

#### *A.1.2 Syntax Rules*

SBGN languages can be thought of as bipartite compound graphs with well-defined syntactic rules governing how objects (or constructs) defined by various SBGNML classes can be connected as well as definitions describing an inclusion hierarchy for how objects can be contained via compartments, complexes, and submaps. A reference implementation[5] of these rules is enumerated and provided using Schematron[6], a rule-based validation language, that operates as an XML stylesheet applicable to SBGN diagrams encoded as XML-based SBGNML documents.

#### *A.1.3 Semantic Rules*

Rules governing the interpretation of SBGN maps are defined in the “Semantic Rules” section of each SBGN language specification. Currently, there is no reference implementation for the rules described in these sections.

#### *A.1.4 Layout Rules*

Rules governing the visual appearance and aesthetics for SBGN maps are defined in the “Layout Rules” section of each SBGN language specification. Currently, there is no reference implementation for the rules described in these sections as aesthetics is a highly subjective area.

[5] https://github.com/sbgn/libsbgn
[6] http://schematron.com

## B Including color / style information

While SBGNML does not formally define classes and attributes that attach color and rendering information to the **Glyph** and **Arc** classes of an SBGN **Map**, the consensus is, to make use of the SBML Level 3 Render Package (Bergmann et al., 2018). This is being done by adding an SBML `renderInformation` element as a child of the `extension` element of a **Map**. The render information object, then defines a list of colors to be used throughout the document, and its list of styles allows to attach these colors to the `id` of children of the **Map**.

The example below defines two colors, "`color_1`" (to be used as the background color for the glyph) and "`black`" (to be used as the outline). They are attached to the glyph with id "`sa5`" via the `idList` attribute `style` element.

```
<?xml version='1.0' encoding='UTF-8' standalone='yes'?>
<sbgn xmlns="http://sbgn.org/libsbgn/0.3">
  <map id="map" language="process description">
    <extension>
      <renderInformation
        xmlns="http://www.sbml.org/sbml/level3/version1/render/version1"
        id="renderInformation">

<listOfColorDefinitions>
          <colorDefinition id="color_1" value="#ccffccff"/>
          <colorDefinition id="black" value="#000000"/>
        </listOfColorDefinitions>

<listOfStyles>
          <style id="example" idList="sa5">
            <g stroke="black" stroke-width="2" fill="color_1"/>
          </style>
        </listOfStyles>
      </renderInformation>
    </extension>
    <glyph id="sa5" class="macromolecule">
      <label text="s5"/>
      <bbox x="90" y="20" w="80" h="40"/>
    </glyph>
  </map>
</sbgn>
```

Where the previous example used the `idList` in the **Style** class to indicate that it applies to a specific **Glyph** with that `id`, the following example uses a different mechanism. This time the `roleList` indicates, that the **Style** applies to glyphs with a `render:objectRole` attribute.

```
<?xml version='1.0' encoding='UTF-8' standalone='yes'?>
<sbgn xmlns="http://sbgn.org/libsbgn/0.3"
  xmlns:render="http://www.sbml.org/sbml/level3/version1/render/version1">
  <map id="map" language="process description">
    <extension>
      <renderInformation
        xmlns="http://www.sbml.org/sbml/level3/version1/render/version1"
        id="renderInformation">

<listOfColorDefinitions>
          <colorDefinition id="color_1" value="#ccffccff"/>
          <colorDefinition id="black" value="#000000"/>
        </listOfColorDefinitions>

<listOfStyles>
          <style id="example" roleList="example_style">
            <g stroke="black" stroke-width="2" fill="color_1"/>
          </style>
        </listOfStyles>
```

```
      </renderInformation>
    </extension>
    <glyph id="sa5" class="macromolecule" render:objectRole="example_style">
      <label text="s5"/>
      <bbox x="90" y="20" w="80" h="40"/>
    </glyph>
  </map>
</sbgn>
```

## C Acknowledgments

Here we acknowledge those people and organizations that assisted in the development of version 0.3 release of the SBGNML specification. We acknowledge contributors that attended workshops and forum meetings or, in some other way, provided input to previous revisions to this effort. Then, we acknowledge the bodies that provided financial support for the development of the standard.

The following list includes members of the SBGN community that have contributed to the development of SBGNML in the past. Their efforts helped shape what SBGNML is today:

- Mirit I. Aladjem
- Sarah E. Boyd
- Emek Demir
- Akira Funahashi
- Hiroaki Kitano
- Nicolas Le Novère
- Yukiko Matsuoka
- Huaiyu Mi
- Stuart L. Moodie
- Herbert M. Sauro
- Falk Schreiber
- Anatoly Sorokin
- Martijn P. van Iersel
- Alice C. Villéger

The UML diagrams were constructed using Visual Paradigm, a leading provider for Business and IT Transformation software solutions.

## D Financial Support

The development of SBGN was mainly supported by a grant from the Japanese *New Energy and Industrial Technology Development Organization* (NEDO, http://www.nedo.go.jp/). The *Okinawa Institute of Science and Technology* (OIST, http://www.oist.jp/), the *AIST Computational Biology Research Center* (AIST CBRC, http://www.cbrc.jp/index.eng.html) the British *Biotechnology and Biological Sciences Research Council* (BBSRC, http://www.bbsrc.ac.uk/) through a Japan Partnering Award, the European Media Laboratory (EML Research gGmbH, http://www.eml-r.org/), and the Beckman Institute at the California Institute of Technology (http://bnmc.caltech.edu) provided additional support for SBGN workshops. Some help was supplied by the *Japan Science and Technology Agency* (JST, http://www.jst.go.jp/) and the *Genome Network Project* of the Japanese Ministry of Education, Sports, Culture, Science, and Technology (MEXT, http://www.mext.go.jp/) for the development of the gene regulation network aspect of SBGN, and from the *Engineering and Physical Sciences Research Council* (EPSRC, http://www.epsrc.ac.uk) during the redaction of the specification. The *German Ministry of Education and Research* (https://www.bmbf.de/) provided support for an SBGN workshop, the redaction of the specification, and development of the SBGNML specification (de.NBI partner project "ModSim" (FKZ: 031L0104A)). Funding from *National Institute of General Medical Sciences (NIGMS)* grant (P41 GM103504), *National Human Genome Research Institute (NHGRI)* grant (U41 HG006623), and *The Scientific and Technological Research Council of Turkey* grant (113E161) was additionally used for work on the specification. Funding from the US National Institutes of Health (NIH, grant number 2R01GM070923-13) supported software development related to SBGN.

## References

Adorno, M., Cordenonsi, M., Montagner, M., Dupont, S., Wong, C., Hann, B., Solari, A., Bobisse, S., Rondina, M. B., Guzzardo, V., Parenti, A. R., Rosato, A., Bicciato, S., Balmain, A., and Piccolo, S. (2009). A Mutant-p53/Smad Complex Opposes p63 to Empower TGF$\beta$-Induced Metastasis. *Cell*, 137(1):87–98.

Balaur, I., Roy, L., Mazein, A., Karaca, S. G., Dogrusoz, U., Barillot, E., and Zinovyev, A. (2020). cd2sbgnml: bidirectional conversion between CellDesigner and SBGN formats. *Bioinformatics*.

Bergmann, F. T., Keating, S. M., Gauges, R., Sahle, S., and Wengler, K. (2018). SBML Level 3 Package: Render, Version 1, Release 1. *Journal of Integrative Bioinformatics*, 15(1).

Biron, P. V. and Malhotra, A. (2000). XML Schema Part 2: Datatypes (W3C Candidate Recommendation 24 October 2000). Available via the World Wide Web at http://www.w3.org/TR/xmlschema-2/.

Bray, T., Paoli, J., Sperberg-McQueen, C., Maler, E., and Yergeau, F. (2004). Extensible Markup Language (XML) 1.0. Available via the World Wide Web at https://www.w3.org/TR/2004/REC-xml-20040204/.

Büchel, F., Rodriguez, N., Swainston, N., Wrzodek, C., Czauderna, T., Keller, R., Mittag, F., Schubert, M., Glont, M., Golebiewski, M., van Iersel, M., Keating, S. M., Rall, M., Wybrow, M., Hermjakob, H., Hucka, M., B Kell, D., Müller, W., Mendes, P., Zell, A., Chaouiya, C., Saez-Rodriguez, J., Schreiber, F., Laibe, C., Dräger, A., and Le Novère, N. (2013). Path2Models: large-scale generation of computational models from biochemical pathway maps. *BMC Systems Biology*, 7(1):116.

Croft, D., O'Kelly, G., Wu, G., Haw, R., Gillespie, M., Matthews, L., Caudy, M., Garapati, P., Gopinath, G., Jassal, B., Jupe, S., Kalatskaya, I., Mahajan, S., May, B., Ndegwa, N., Schmidt, E., Shamovsky, V., Yung, C., Birney, E., Hermjakob, H., D'Eustachio, P., and Stein, L. (2011). Reactome: a database of reactions, pathways and biological processes. *Nucleic Acids Research*, 39:D691–D697.

Czauderna, T., Klukas, C., and Schreiber, F. (2010). Editing, validating and translating of SBGN maps. *Bioinformatics*, 26(18):2340–2341.

Czauderna, T. and Schreiber, F. (2017). Information Visualization for Biological Data. In Keith, J. M., editor, *Bioinformatics: Volume II: Structure, Function, and Applications*, volume 1526 of *Methods in Molecular Biology*, pages 403–415. Springer.

Dennis, A., Wixom, B. H., and Tegarden, D. (2015). *Systems Analysis and Design: An Object-Oriented Approach with UML 5th Edition*. Wiley.

Hucka, M., Bergmann, F. T., Chaouiya, C., Dräger, A., Hoops, S., Keating, S. M., König, M., Le Novère, N., Myers, C. J., Olivier, B. G., Sahle, S., Schaff, J. C., Sheriff, R., Smith, L. P., Waltemath, D., Wilkinson, D. J., and Zhang, F. (2019). Systems Biology Markup Language (SBML) Level 3 Version 2 Core release 2. *Journal of Integrative Bioinformatics*, 16(2):1.

Junker, A., Rohn, H., Czauderna, T., Klukas, C., Hartmann, A., and Schreiber, F. (2012). Creating interactive, web-based and data-enriched maps with the Systems Biology Graphical Notation. *Nature Protocols*, 7(3):579–593.

King, Z. A., Dräger, A., Ebrahim, A., Sonnenschein, N., Lewis, N. E., and Palsson, B. O. (2015). Escher: A web application for building, sharing, and embedding data-rich visualizations of biological pathways. *PLoS Computational Biology*, 11(8):e1004321.

Kuperstein, I., Bonnet, E., Nguyen, H.-A., Cohen, D., Viara, E., Grieco, L., Fourquet, S., Calzone, L., Russo, C., Kondratova, M., Dutreix, M., Barillot, E., and Zinovyev, A. (2015). Atlas of Cancer Signalling Network: a systems biology resource for integrative analysis of cancer data with Google Maps. *Oncogenesis*, 4:e160.

Mi, H., Huang, X., Muruganujan, A., Tang, H., Mills, C., Kang, D., and Thomas, P. D. (2017). PANTHER version 11: expanded annotation data from Gene Ontology and Reactome pathways, and data analysis tool enhancements. *Nucleic Acids Research*, 45:D183–D189.

Mi, H., Schreiber, F., Moodie, S., Czauderna, T., Demir, E., Haw, R., Luna, A., Le Novère, N., Sorokin, A., and Villéger, A. (2015). Systems Biology Graphical Notation: Activity Flow language Level 1 Version 1.2. *Journal of Integrative Bioinformatics*, 12:265.

Mullis, K., Faloona, F., Scharf, S., Saiki, R., Horn, G., and Erlich, H. (1986). Specific Enzymatic Amplification of DNA In Vitro: The Polymerase Chain Reaction. In *Cold Spring Harbor Symposia on Quantitative Biolology*, volume 51, pages 263–273.

Novère, N. L., Hucka, M., Mi, H., Moodie, S., Schreiber, F., Sorokin, A., Demir, E., Wegner, K., Aladjem, M. I., Wimalaratne, S. M., Bergman, F. T., Gauges, R., Ghazal, P., Kawaji, H., Li, L., Matsuoka, Y., Villéger, A., Boyd, S. E., Jouraku, A., Kim, S., Kolpakov, F., Luna, A., Sahle, S., and Schmidt, E. (2009). The Systems Biology Graphical Notation. *Nature Biotechnology*, 27(8):735–742.

Pon, A., Jewison, T., Su, Y., Liang, Y., Knox, C., Maciejewski, A., Wilson, M., and Wishart, D. S. (2015). Pathways with PathWhiz. *Nucleic Acids Research*, 43:W552–W559.

Rodchenkov, I., Babur, O., Luna, A., Aksoy, B. A., Wong, J. V., Fong, D., Franz, M., Siper, M. C., Cheung, M., Wrana, M., Mistry, H., Mosier, L., Dlin, J., Wen, Q., O'Callaghan, C., Li, W., Elder, G., Smith, P. T., Dallago, C., Cerami, E., Gross, B., Dogrusoz, U., Demir, E., Bader, G. D., and Sander, C. (2020). Pathway Commons 2019 Update: integration, analysis and exploration of pathway data. *Nucleic Acids Research*, 48:D489–D497.

Rougny, A., Touré, V., Moodie, S., Balaur, I., Czauderna, T., Borlinghaus, H., Dogrusoz, U., Mazein, A., Dräger, A., Blinov, M. L., Villéger, A. C., Haw, R., Demir, E., Mi, H., Sorokin, A., Schreiber, F., and Luna, A. (2019). Systems Biology Graphical Notation: Process Description language Level 1 Version 2.0. *Journal of Integrative Bioinformatics*, 16(2).

Sari, M., Bahceci, I., Dogrusoz, U., Sumer, S. O., Aksoy, B. A., Babur, O., and Demir, E. (2015). SBGNViz: A Tool for Visualization and Complexity Management of SBGN Process Description Maps. *PloS one*, 10:e0128985.

Schreiber, F., Colmsee, C., Czauderna, T., Grafahrend-Belau, E., Hartmann, A., Junker, A., Junker, B. H., Klapperstück, M., Scholz, U., and Weise, S. (2012). MetaCrop 2.0: managing and exploring information about crop plant metabolism. *Nucleic Acids Research*, 40:D1173–D1177.

Sorokin, A., Le Novère, N., Luna, A., Czauderna, T., Demir, E., Haw, R., Mi, H., Moodie, S., Schreiber, F., and Villéger, A. (2015). Systems Biology Graphical Notation: Entity Relationship language Level 1 Version 2. *Journal of Integrative Bioinformatics*, 12:264.

Touré, V., Le Novère, N., Waltemath, D., and Wolkenhauer, O. (2018). Quick tips for creating effective and impactful biological pathways using the Systems Biology Graphical Notation. *PLoS Computational Biology*, 14:e1005740.

Touré, V., Mazein, A., Waltemath, D., Balaur, I., Saqi, M., Henkel, R., Pellet, J., and Auffray, C. (2016). STON: exploring biological pathways using the SBGN standard and graph databases. *BMC Bioinformatics*, 17:494.

van Iersel, M. P., Villéger, A. C., Czauderna, T., Boyd, S. E., Bergmann, F. T., Luna, A., Demir, E., Sorokin, A., Dogrusoz, U., Matsuoka, Y., Funahashi, A., Aladjem, M. I., Mi, H., Moodie, S. L., Kitano, H., Le Novère, N., and Schreiber, F. (2012). Software support for SBGN maps: SBGN-ML and LibSBGN. *Bioinformatics*, 28:2016–2021.
